# Athletes’ Self-Assessment of Urine Color Using Two Color Charts to Determine Urine Concentration

**DOI:** 10.3390/ijerph18084126

**Published:** 2021-04-13

**Authors:** Floris C. Wardenaar, Daniel Thompsett, Kaila A. Vento, Kathryn Pesek, Dean Bacalzo

**Affiliations:** 1 College of Health Solutions, Arizona State University, Phoenix, AZ 85004, USA; djthom12@asu.edu (D.T.); kvento@asu.edu (K.A.V.); kpesek@asu.edu (K.P.); 2 Herberger Institute for Design and the Arts, Arizona State University, Tempe, AZ 85281, USA; dbacalzo@kean.edu; 3 Michael Graves College, Wenzhou-Kean University, No.88 Daxue Road, Ouhai District, Wenzhou 325060, China

**Keywords:** self-reporting, hydration status, osmolality, USG, accuracy, validation

## Abstract

Our objective was to determine self-reported accuracy of an athletic population using two different urine color (Uc) charts (8-color vs. 7-color Uc chart). After approval by the Institutional Review Board, members of an athletic population (n = 189, 20 (19–22) year old student- or tactical athletes and coaches, with n = 99 males and n = 90 females) scored their Uc using two charts. To determine the diagnostic value of Uc, results were compared with urine concentration (osmolality and urine specific gravity, USG). Uc was scored slightly darker with the 8-color vs. 7-color Uc chart (2.2 ± 1.2 vs. 2.0 ± 1.2, respectively, *p* < 0.001), with a moderate correlation between charts (*r* = 0.76, 95% CI: 0.69–0.81). Bland-Altman analysis showed a weak reporting bias (*r* = 0.15, *p* = 0.04). The area under the curve for correct urine sample classification ranged between 0.74 and 0.86. Higher accuracy for both methods was found when Uc scores were compared to USG over osmolality, indicated by 4.8–14.8% range in difference between methods. The optimal Uc cut-off value to assess a low vs. a high urine concentration for both Uc charts varied in this study between 1 and ≤2 while accuracy for charts was similar up to 77% when compared to USG.

## 1. Introduction

Introduced 25 years ago, urine color (Uc) correlates with body water deficits [1] and changes in body water [2]. As a clinical measure of urine concentration, and thus hydration status, urine osmolality and urine specific gravity (USG) have higher sensitivity than Uc [3]. However, Uc is an inexpensive, non-invasive, and easily-performed means of assessing hydration status [3,4,5], even for the untrained individual [4,6,7], and therefore it can serve as a simple tool to identify if athletes need to drink more. The traditional 8-color Uc chart developed by Armstrong et al. (1994), or modifications of this chart, can be found in many athletic facilities. However, the accuracy of self-reporting Uc based on multi-shade urine color charts needs validation [8], for example in athletic populations, allowing health professionals to better educate their athletes on the use and interpretation of multiple shade color charts [9].

Only three studies have described the validity of Uc self-assessment to determine urine concentration. Two reported the accuracy of Uc scoring in a population of adults and a group of children [4,6]. A recent study described a novel way athletes can score Uc directly from the toilet bowl [7]. Despite differences in methods, these studies reported similar numbers for correctly classifying low vs. high urine concentrations, based on calculated area under the curve (AUC) values, ranging from 0.67–0.78.

There is substantial variation in Uc charts, as they come in many different colors and sizes, so there can be differences in their comparability and therefore their accuracy. Many charts available on the internet were probably derived from the traditional 8-color Uc chart, which was based on the classification and ranking based on the color of a large number of urine samples [1]. The validity of Uc scoring with color charts has been determined when performed by investigators [2,4,6], however, these studies employed different containers, fluid volumes, and lighting conditions, making it difficult to compare outcomes. The idea is that Uc charts’ cut-off values indicate which urine sample has a low vs. high urine concentration, but this depends on methods under which Uc is scored [4]. Cut-off values should therefore be used as a sliding scale, not as a one-size-fits-all approach. The Beer–Lambert law states that light absorbance is equal to the product of (1) the concentration of the solution the light passes through it and the material of the container, (2) solution depth, and (3) the absorption coefficient [4]. Thus, perceived color is influenced by the sample container (glass vs. plastic) and the urine cup’s diameter [4]. In combination with the color and intensity of light, these factors will affect light absorption, influencing the rater’s response. Therefore, studies examining validity of athlete Uc assessment using multiple color charts, while standardizing for confounding factors such as cup size, material, and light intensity, are non-existent.

As there is a lack of insight in the validation of athlete self-reporting Uc to identify urine with a low vs. high concentration, as well as an insufficient understanding of the comparability of Uc charts, our objective was to determine the accuracy of urine color (Uc) scoring by an athletic population between a traditional 8-color Uc chart and a newly developed 7-color Uc chart. The aims were as follows: (a) to determine differences in scoring Uc for two different charts; and (b) to investigate the diagnostic ability and the optimal Uc cut-off value of the two Uc charts to assess a low vs. high urine concentration, based on a pre-defined USG and urine osmolality cut-off value.

## 2. Materials and Methods

### 2.1. Participants

An athletic population of 189 university National Collegiate Athletic Association (NCAA) Division I athletes in the USA, student club athletes, coaches, and tactical athletes (recruits of the Army Reserve Officer Training Corps, ROTC) (52% male, 22.3 ± 1.6 years) participated. They reported being in good self-reported health and not using medications that could influence hydration status. Dietary supplement intake was checked, but participants using them were not excluded. Data were collected as part of a broader project that included the validation of a urine color scale to assess urine color directly from the toilet bowl [7]. A Power of Sign Test revealed a 98% power to detect the distribution of samples below (58%) and above (42%) a selected USG threshold of 1.020 defining low vs. high urine concentration, respectively (with *p* = 0.50 at *α* = 0.05) based on this sample (n = 189). The Institutional Review Board of Arizona State University approved the study (STUDY00010071). Participants gave written consent and, except coaches, received a $15 digital incentive after participation.

### 2.2. Urine Color Charts

To be able to better understand the accuracy of Uc self-assessments in an athletic population, and to generalize results, we suggest evaluation of a mix of color charts derived from different methods, i.e., charts based on ranking urine colors vs. charts based on urine concentration-based color categories. As many Uc charts are available, this approach will help to better understand the validity of urine color charts that were constructed based on different mechanisms.

#### 2.2.1. Development of the New 7-Color Uc Chart

The new 7-color Uc developed by Wardenaar and Bacalzo in 2019 was based on a selection of 96 previously collected urine samples [10]. The Uc categories were based on the following USG ranges with (n) indicating the number of urine samples in each category: <1.017 (n = 20), 1.017–1.021 (n = 15), 1.022–1.023 (n = 13), 1.024–1.026 (n = 24), 1.027–1.028 (n = 9), 1.029–1.031 (n = 11), and >1.031 (n = 4). This resulted in a range of 7 colors, with a very low concentration (score 1) to a very high concentration (score 7), consistent with the categories for hydration status suggested by Armstrong et al. (2010). A collective sample reflecting a urine concentration category was produced by combining an equal amount of each sample within the urine concentration category. This was done for each concentration category. Each newly produced color sample was aliquoted in a 30 mL tube without caps (30 mL free-standing Evergreen centrifuge tube, Caplugs, Buffalo, NY, USA), and placed in our specially designed scoring box. Tubes were lighted underneath by white LED flashlight (Ozark Trail, Ozark, Betonfille, AR, USA) with 1848 lux measured using a foot-candle lux meter (Extech 407026, Extech Instruments, Waltham, MA, USA) at the Tungsten/Daylight setting. Photos were taken of each collective sample with a digital single-lens reflex (SLR) camera, Canon EOS Rebel T4i with a Canon Zoom Lens EF-S 18–55 mm f/3.5–5.6 (Canon Inc., Ōta, Tokyo, Japan). The camera was positioned 35.5 cm (14 inches) from the sample and placed behind a round 3.05 cm (1.2 inch) opening showing the sample against a white backdrop. White balance was set by taking a photo of a freestanding tube with 30 mL demineralized water. After digital photos were taken, the white color reference was confirmed using Adobe Photoshop (Adobe Inc., San Jose, CA, USA) by identifying the whitest white and the blackest black. Then on-screen urine colors (Red-Green-Blue/RGB) were translated to a print color system. This resulted in the following Pantone Matching System (X-rite, Grand Rapids, MI, USA), also called PMS colors (with hexadecimal (HEX) codes 0 short RGB color codes in parentheses), printed from left to right in rectangles size 5.1 × 7.6 cm (2 × 3 inch): Color 1: 7499 C (F1E6B2); color 2: 600 C (F1EB9C); color 3: 602 C (F0E87B); color 4: 603 C (EDE04B): color 5: 605 C (E1CD00); color 6: 7758 C (D4C304) and color 7: 124 C (EAAA00). Prints were made on a white paper measuring 43.2 × 27.9 cm (17 × 11 inch) with a gloss finish.

#### 2.2.2. Remaking a Traditional 8-Color Chart

We used the colors reported on the 8-color chart described in the Dictionary of Color [11] as reported previously [1]: color 1 17/B1; color 2 9/H1; color 317/J1; color 4 17/L1; color 5 9/I3; color 69/L3; color 712/K6 and color 8 23/L1. These colors were digitally transferred to HEX codes and matte paints were selected with this exact code. This resulted in the following HEX codes and paint colors in parenthesis: color 1 FFFDD8 (DE 5407 Pumpkin seed (I), Interior velvet L Base); color 2 FFFBA8 (DE 5402 Lemon slice (I), Interior velvet L Base); color 3 FCE974 (DE 5417 Dandelion (I) (LH), Interior velvet M Base); color 4 FFBA00 (Yellow Finch 19D-5, Premium interior satin enamel); color 5 FFCE79 (DE 5290 Apricot glow (I), Interior velvet M Base); color 6 EAC853 (DE 5424 Yellow brick road (I) (LH), Interior velvet U Base); color 7 E1C161 (DE 5432 Candelabra (I), Interior velvet U Base); and color 8 898253 (DE 5496 Aged eucalyptus, Interior velvet U Base). All paints were Dunn–Edwards paints (Dunn—Edwards Corporation, Los Angeles, CA, USA), except for color 4 being a Clark + Kensington paint (Ace Hardware Corporation, Oak Brook, IL, USA). Each color was then painted onto wooden 2.5 × 15.2 cm (1 × 6 inch) rectangles and glued onto a 48.3 × 30.5 cm (19 × 12 inch) matte whiteboard with an equal 2.5 cm (1-inch) distance apart.

### 2.3. Uc Scoring Box

**Figure 1:**
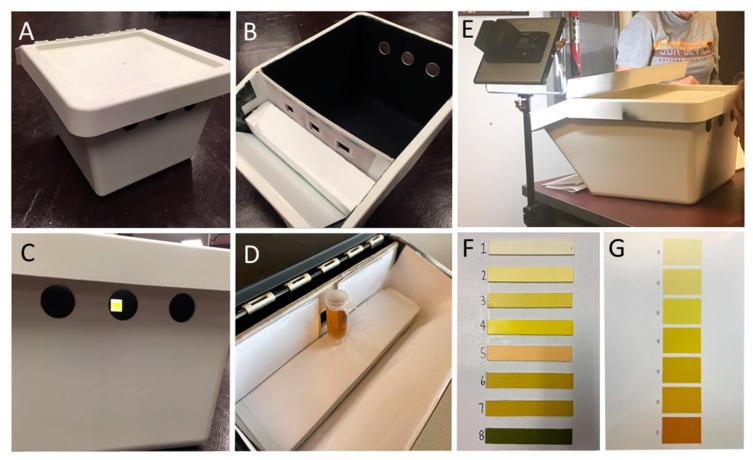
Urine color scoring box and pictures of the Uc charts used during this study. (**A**): The box (Sortera 37.9 L (10 gal), IKEA, Almhult, Sweden) was converted to score urine samples by adding three holes in the front; (**B**): The box was painted black and a white wall was positioned at the end of the box, with three square holes of 2.2 × 2.2 cm (1 × 1 inch) in it; (**C**): For the purpose of this study, only the middle hole was used to score one urine sample at a time while the other holes were covered; (**D**): The urine sample, covered with transparent foil, was positioned behind the middle square hole in front of a white backdrop while the left and right squared holes were covered; (**E**): Athletes were seated while scoring the urine sample, with the sample lighted from the left site using a 28-watt color adjustable lamp providing an intensity of ~1650 lux at full power and light color set to white (NL480, Neewer, Shenzen, China) while color charts are on the right of the scoring box; (**F**): A picture of the 8-color Uc chart remake with colors adapted from Armstrong et al. (1994); (**G**): A picture of the 7-color Uc chart developed by Wardenaar and Bacalzo in 2019.

A box was constructed to standardize the participants’ scoring (Figure 1). The box was black inside and was positioned on a white table, with the urine sample tube placed against a white backdrop 35.5 cm (14 inches) from a peephole 2.54 × 2.54 cm (1 × 1 inch) cut in the front of the box. To control lighting, a 28-watt color adjustable lamp providing an intensity of ~1650 lux at full power and light color set to white (NL480, Neewer, Shenzen, China) was placed on the left side of the box at the height of the sample, the scoring was done in a well-lit room directly under a 3-light fluorescent parabolic troffer (420 lux) built into the ceiling.

### 2.4. Procedures

#### 2.4.1. Urine Color Scoring

Black cups were provided to each participant and voiding time was registered to the nearest second and noted on the container lid. If their urine sample was not collected at our site, participants were instructed to perform Uc testing within four hours of collection. Participants handed in their urine sample and recorded their study ID, time collected (first morning or later), voiding duration (sec), sex, age (years), and type of athlete affiliation. Body height (cm) (Seca 213 portable stadiometer, Hamburg, Germany) and body weight (kg) (Seca 803 digital scale, Hamburg, Germany) were measured. A research team member prepared three separate 1.5 mL tubes for measuring urine osmolality, as well as a 30 mL urine sample (30 mL free-standing Evergreen centrifuge tube, Caplugs, Buffalo, NY, USA) for Uc scoring. Each sample was covered using clear Parafilm (Laboratory Film, Bemis Company Inc., Neenah, WI, USA) to seal and prevent color distortion. Participants were then instructed to look into the box and score their sample using the two color charts. The 7- and 8-color charts were alternately used for scoring by participants based on the chart that their predecessor scored first.

#### 2.4.2. Urinalysis

Urine collection cups were measured before and after collection of the sample on a precision scale with 0.1 g accuracy (PT 1400, Sartorius AG, Göttingen, Germany), and so true urine weight could be obtained with the assumption that grams of urine could be translated to milliliters. After Uc was scored, both the 1.5 mL as the 30 mL urine samples were stored at 5 °C until further analysis for urine concentration. Urine can be stored at fridge temperature (5 °C) for 5–7 days without a change in concentration [12].

Urine specific gravity was measured in fresh urine samples (stored no longer than five days in the refrigerator) using a USG refractometer pen (Pen–Urine S.G., Atago, Tokyo, Japan) at a sample temperature of 20 °C. Each measurement was performed twice; in case a variance larger than 0.0005 was detected between the two measurements, a third measurement was added and the median was calculated. Duplicate measurements were performed to calculate mean urine osmolality (with sample CV 0.17 ± 0.18) in fresh urine samples (stored no longer than seven days at a temperature of at 5 ± 1 °C) using freezing point depression (A2O Osmometer, Advanced Instruments, Norwood, MA, USA) [7].

### 2.5. Statistical Analyses

Personal characteristics (height: m, weight: kg), urine volume (mL), urine voiding time (sec), USG, osmolality (mmol·kg^−1^), and Uc were reported as medians and interquartile range, or when appropriate as mean ± standard deviation (sd). Associations based on Spearman correlation coefficients *r* (including 95% CI using Fisher’s Z transformation) are reported for urine color, -voiding time, -volume, USG, and osmolality.

To address aim (a), to determine differences in scoring Uc for two different charts, mean differences were analyzed via Mann–Whitney U tests. Spearman’s correlation coefficient tests correlated Uc scores against urine concentration. A Bland—Altman plot was produced comparing Uc scores from 1–7 (as score 8 was not reported using the 8-color Uc chart) to evaluate charts against each other. To assess whether Bland–Altman results were biased between charts for scoring Uc lighter, similar or darker, a Spearman correlation coefficient was calculated using the Bland–Altman results from the y-axis (difference between reported Uc outcomes) vs. x-axis (means of both outcomes). The level of agreement is expressed as M_(difference)_ ± 1.96 SD_(difference)_. Statistical significance was accepted at *p* ≤ 0.05.

To address aim (b), to determine the color charts’ diagnostic ability to assess underhydration based on the correct classified urine samples for urine concentration, receiver operating characteristics (ROC) curves were calculated. This resulted in the calculation of the area under the curve (AUC), sensitivity, specificity, optimal Uc threshold score and accuracy of correct Uc scores (%) in classifying urine concentration, as well as true positive (TP), true negative (TN), false positive (FP), and false negative (FN) scores. When interpreting the area under the curve an AUC ≥ 0.90 is considered excellent, 0.80–0.89 is considered good, while an AUC of 0.70–0.79 is to be considered fair. Sensitivity and specificity scores are preferred to be above 0.80. Sensitivity is defined as the number of true positive (TP) scores suggesting underhydration, divided by the sum of TP and false-negative (FN) scores [13]. Specificity is defined as the number of true negatives (TN) divided by the sum of false positives (FP) and TN. To classify urine samples with a low vs. high concentration, the optimal Uc threshold score to predict underhydration was determined from the area under the curve (AUC) using the max approach of the sensitivity and specificity matching the selected urine concentration cut-off values for USG < 1.020 [14] and osmolality <800 mmol·kg^−1^ [15]. As dietary supplement use may influence Uc scoring, a stratified analysis was performed to assess the accuracy of Uc scoring splitting supplement users and non-users.

## 3. Results

As shown in Table 1, of the 189 participants, most were White (n = 105), most were student-athletes (n = 132), and about half were female. Visiting Chinese coaches (n = 24) were substantially older (37 years, 34 to 39) than the rest of the population (20 years, 19–22).

The overall median urine volume was 248 mL (138 to 391), 58.7% of the participants delivered a first morning sample, and the median voiding duration was 16 s (11 to 25). The total sample had a median osmolality of 705 mmol·kg^−1^ (465–930) and urine specific gravity of 1.019 (1.013 to 1.024). Correlations between urine osmolality and urine specific gravity ranged between *r* = 0.80–0.97, and all correlations reported in Table 1 were significant (*p* < 0.001).

**Figure 2:**
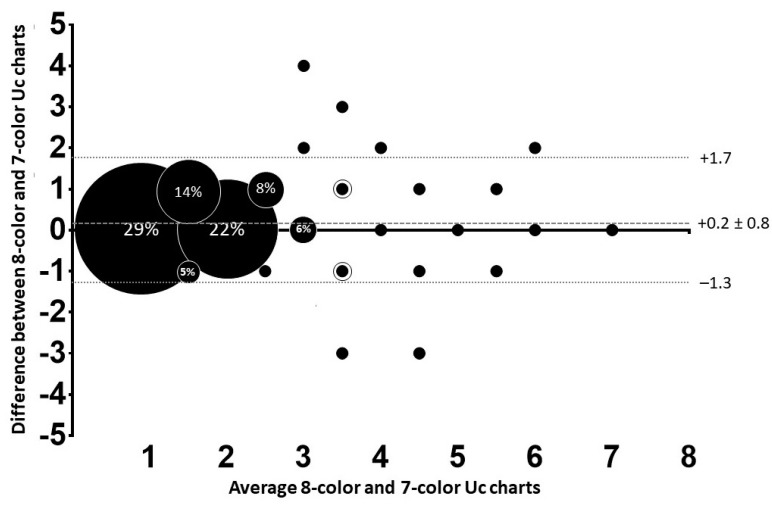
Bland–Altman comparison between 8-color and 7-color Uc charts (n = 189). The 8-color Uc chart remake was based on colors adapted from Armstrong et al. (1994) and the 7-color Uc chart developed by our lab, the Athleat Field Lab; by Wardenaar and Bacalzo in 2019. The size of the bubbles indicates the percentage of urine color scores, small black dots represent 1% values, and circled dots represent 2–3% values. The average reporting bias is indicated by the dark grey dotted line, and the 95% limits of agreement are indicated by the light grey dotted lines.

To answer aim (a), to determine differences in scoring Uc between both charts, the 8-color Uc chart scored Uc significantly darker than the 7-color Uc chart (2.2 ± 1.2 vs. 2.0 ± 1.2, respectively, *p* < 0.001). There was a moderate correlation between Uc charts (*r* = 0.76, 95% CI: 0.69–0.81). The Bland–Altman plot (Figure 2) shows that 60.5% of the scores of the 8-color and 7-color Uc charts were similar. When comparing scores ± 1 color shade, similarity was 96.5%. There was a significant weak reporting bias (*p* = 0.04) indicated by *r* = 0.15 between both Uc charts, indicating that the 7-color Uc chart resulted in slightly lighter scoring than the 8-color Uc chart.

As to aim (b), determining the diagnostic ability of the two Uc charts vs. urine concentration measures, data in Table 2 suggest the diagnostic ability of the 8- and 7-color charts on a group level is fair in relation to osmolality (0.76 and 0.74, respectively), and good in relation to USG (0.86 and 0.83, respectively). Sensitivity was low and specificity was high when Uc scores were related to osmolality. USG values for sensitivity and specificity were less consistent. For the 8- and 7-color Uc charts, the accuracy of Uc against urine concentration was higher for USG (77.2% and 76.7%, respectively) compared to osmolality (63.5% and 66.1%, respectively). The optimal cut-off Uc, needed to accurately classify urine samples for a low vs. high concentration, was 1 when both Uc chart scores were compared with osmolality. When compared to USG, the 8-color Uc chart resulted in a cut-off Uc value ≤2 while the 7-color Uc chart had the best fit for a Uc of 1 to detect low vs. high urine concentration.

Finally, no clear differences for the AUC were seen when stratified analysis was performed for sex. Both men and women reported consistently with the AUC reported on group level. An additionally stratified analysis assessing the AUC and the correct classification of urine samples for participants using dietary supplements (n = 28) vs. those not using them (n = 161) showed no substantial difference between groups. The AUC for supplement users (0.85) was actually higher for both Uc charts vs. non-users (0.82 for the 8-color Uc chart, and 0.80 for the 7-color Uc chart). The accuracy of correct scored urine samples for dietary supplement user and non-users was slightly reversed for the 8-color Uc chart (71.4% vs. 74.5% accuracy, respectively), but not for the 7-color Uc chart (75.0% vs. 73.9% accuracy, respectively).

## 4. Discussion

This study is the first to report the validity of a self-assessment of traditional multi-shade Uc assessment in an athletic population using different Uc charts to classify low vs. high urine concentration. This knowledge will help athletes determine whether to increase fluid consumption on a daily basis. Additionally, the results will inform health professionals about the strengths and limitations of this method. The diagnostic ability of the charts expressed by the AUC and the accuracy of correct classified urine samples was fair (8-color Uc chart) to good (7-color Uc chart). Depending on the type of chart used, athletes may report slightly darker Uc scores when scoring urine samples based on the 8-color vs. the 7-color Uc chart. Finally, the self-reported accuracy was almost 10% higher when Uc scores were compared to a USG cut-off value of 1.020 vs. a urine osmolality cut-off value of 800 mmol·kg^−1^.

The osmolality based AUC reported in this study was 0.76 for 8-color Uc chart and 0.74 for 7-color Uc chart, similar to self-reported values (0.67–0.78) in children [4]. The USG-based AUC was 0.74 for 8-color Uc chart and 0.83 for 7-color Uc chart, which is equal to or higher than an earlier USG value of 0.73 self-reported in females [6]. Finally, a similar AUC for Uc scoring was found in a recently validated lavatory method assessing urine color from the toilet bowl using diluted color shades, with osmolality (0.73) and USG (0.76) [7]. This suggests that an AUC between 0.74–0.86 is to be expected from untrained individuals scoring the color of urine sample regardless of the chart method used. Overall, laypersons may report slightly lower AUC values than trained investigators reporting a lab technician based AUC of 0.96 [16]. This suggests that when properly trained, athletes can potentially improve the accuracy of their scores.

Significantly more “Uc 2” scores were recorded for the 7-color Uc chart vs. more “Uc 3” scores for the 8-color chart, which could be due to some small color differences in construction between charts. Our relatively small tubes (30 mL) exposed to bright LED light, equal to ~1650 lux, are likely to report 1–2 shades lower cut-off values than previously reported based on samples scored in a “well lit” room [1,2,4]. At this light intensity, both charts reported relatively similar results, but we could speculate if charts are comparable when used under different light conditions, as Figure 1F,G clearly show visual differences between the darker color panels on the charts. Overall, when focusing on the collected results the study shows that, despite minor differences in reporting between the two Uc charts, results for both charts are comparable.

Earlier studies reported optimal cut-off values for 8-color Uc charts between ≤3 [4] and ≤5 [16], while the current study reported values of 1 and ≤2. Based on the current study results, coaches and athletic training staff should inform their athletes that multi-shade Uc charts should be viewed as a sliding scale rather than a single cut-off value depending on the light conditions in which the urine sample it scored.

There was a difference in the accuracy of classifying samples for hypohydration between osmolality and USG. This difference could be the result of a slight mismatch between the selected cut-off values. The selected cut-off value for osmolality (800 mmol·kg^−1^) marked the suggested onset of dehydration, comparable to a USG value of 1.020 based on 24-hour urine collections [14]. Whereas others used a slightly lower cut-off of 700 mmol·kg^−1^ to define hypohydration [17]. Despite the difference in cut-off values for osmolality, Hew–Butler et al. (2018) also used a 1.020 USG cut-off. We suggest the differences in Uc scoring accuracy is likely the result of the two cut-off values diagnosing slightly different hydration states, i.e., USG assessing a lower non-clinical level of hypohydration and the somewhat higher urine osmolality concentration assessing a more progressed form of non-clinical hypohydration.

Although the literature is not consistent about the impact of dietary supplements on urine color scoring [18,19], there is some evidence that riboflavin (B2) influences Uc scoring in a negative way [19]. We found no substantial differences between for correctly classifying low vs. high urine concentrations, based on the calculated AUC for samples provided by supplement users and non-users.

This study was not without its limitations. The number of color shade panels between charts were different, but none of the samples was scored color 8 suggesting that this was not a large limitation while comparing both charts during this data collection. There was no standardization of fluid intake and spot urine samples were collected at various times during the day. Sport morning samples are known to have a somewhat higher concentration than 24-hour urine collections [20,21]. On the other hand, the majority of the samples were collected in the afternoon hours, with urine concentrations closely related to 24-hour urine collections [20,21,22]. Additionally, Uc and urine concentration can be influenced by acute rehydration strategies after practice [23], which could lead to a mismatch between Uc and urine concentration due to acute dilution of the urine. We did not determine time between the last practice in relation to urine collection. It is also likely that the study was impacted by recruitment bias, triggering mainly those interested in assessing their hydration status. Despite a diverse population, the distribution was somewhat skewed with largest part of the population existing of White student-athletes, with fewer Black, Hispanic, and other participants. Finally, this validation was performed in a controlled setting, therefore generalizations towards other use of Uc charts in a different setting need to be made with caution.

Considerations for practitioners include that a high urine concentration is associated with underhydration, a phenomenon in which a low water intake is associated with high vasopressin levels and urine concentration, without bodyweight change or a sensation of thirst [24]. The assessment of Uc can help to identify athletes with a low vs. high fluid intake [25]. This is a simple assessment that can be used in multiple settings, before regular practice, at home, or while travelling. Additionally, hydration assessment can be of use when assessing body composition such as bioimpedance measurements [26,27], or when performing exercise testing [28] allowing for a better standardization of measurements. Despite the small significant difference in reporting Uc, no real practical difference exists between the two validated Uc scales when looking at the total number of correctly classified urine samples (up to 77%). When looking deeper into this misclassification, the 7-color Uc chart showed a much lower number of false-positive classifications (6%) than the 8-color chart (21%). This is an important difference, because false-positive classifications as a result of a light urine color score, while the concentration was actually above the selected urine concentration cut-off value, would likely not prompt an athlete to increase fluid intake. At the same time, the numbers for false-negative classifications were reported in reverse, resulting in a larger number of athletes being prompted to drink by the 7-color Uc chart. This highlights the importance of educating athletes on the proper timing of hydration and rehydration strategies, including advice about drinking volume to ensure safe drinking practices [9]. Preferably, the assessment of Uc should be combined with other methods to allow for a better detection of a suboptimal hydration status [9]. A good example is that a combined assessment of urine color and urine void frequency during a 24-hour period results in a 97% diagnostic ability for underhydration [29].

## 5. Conclusions

The results suggest that the use of two multi-shade Uc charts (one 8-color and one 7-color), regardless of the difference in method for constructing their color shade panel, is similar to Uc self-assessments by athletes. A greater classification accuracy for low vs. high urine concentration occurs, up to 77% correct classified samples, when Uc scoring is compared to urine specific gravity rather than urine osmolality.

## Figures and Tables

**Table 1:** Participant and urine sample characteristics.

	Age (y)	Height (m)	Weight (kg)	Urine Volume (mL)	Urine Concentration			First Morning Sample (%)	Voiding Duration
					Osmolality (mmol·kg^−1^)	USG	r		(sec.)
Total (n = 189)	20 (19–22)	1.73 (1.67–1.79)	70.0 (63.0–79.0)	248 (137–391)	705 (456–930)	1.019 (1.013–1.024)	0.89 *	58.7	16 (11–25)
Sex									
Male (n = 99)	20 (19–22)	1.77 (1.73–1.81)	75.8 (69.2–84.0)	273 (158–437)	741 (505–938)	1.020 (1.013–1.024)	0.80 *	62.6	18 (12–29)
Female (n = 90)	20 (19–21)	1.68 (1.64–1.73)	64.4 (58.0–72.8)	231 (123–366)	655 (382–884)	1.017 (1.011–1.023)	0.97 *	54.4	13 (10–21)
Race and ethnicity									
Black (n = 22)	20 (18–21)	172 (167–176)	68.1 (62.8–74.8)	174 (107–278)	732 (499–1017)	1.020 (1.015–1.028)	0.88 *	13.6	12 (7–17)
White (n = 105)	20 (19–21)	173 (168–179)	70.4 (64.5–79.6)	248 (140–391)	722 (388–931)	1.019 (1.010–1.024)	0.86 *	60.0	17 (11–25)
Hispanic (n = 29)	19 (19–21)	170 (165–179)	67.3 (58.1–77.2)	206 (123–340)	656 (465–932)	1.017 (1.013–1.024)	0.90 *	51.7	13 (9–17)
Other (n = 33)	34 (22–39)	174 (167–180)	73.0 (60.4–81.7)	358 (197–555)	661 (472–859)	1.018 (1.013–1.023)	0.95 *	90.9	28 (17–37)
Exercise level									
Student athlete (n = 132)	20 (19–21)	172 (167–178)	68.7 (62.4–78.4)	217 (122–364)	724 (459–941)	1.019 (1.012–1.025)	0.86 *	41.7	13 (10–21)
Army ROTC (n = 33)	19 (18–21)	175 (166–180)	70.6 (65.5–78.8)	281 (175–424)	616 (385–860)	1.016 (1.012–1.023)	0.98 *	97.0	20 (14–29)
Coach (n = 24)	37 (34–39)	174 (168–180)	73.3 (60.8–84.7)	400 (329–620)	663 (471–834)	1.019 (1.014–1.022)	0.91 *	100	34 (22–40)

All continuous variables are provided as median and interquartile range in parentheses. All Spearman correlation coefficients between osmolality and USG were significant, * = *p* < 0.001. The counterpart of first morning sample are samples collected at any other time during the day. Abbreviations: Army ROTC = Army Reserve Officers’ Training Corps; USG = urine specific gravity.

**Table 2:** Urine color scores, urine concentrations, AUC, accuracy of classification, and receiver operating characteristics (ROC)-based Uc cut-off values scored by participants based on n = 189 urine samples for two different Uc charts.

Category	Urine Concentration Markers			Receiver Operator Characteristics								
	Uc	Min-Max	r (Uc vs. Concentration)	AUC	Sensitivity	Specificity	TP	TN	FP	FN	Accuracy	Cut-off Uc
8-color chart vs. Osm	2 (1–3)	1–7	0.65 * (0.56–0.73)	0.76	47.5%	91.3%	57	63	6	63	63.5%	1
8-color chart vs. USG	2 (1–3)	1–7	0.74 * (0.67–0.80)	0.86	95.1%	56.3%	97	49	38	5	77.2%	≤2
7-color chart vs. Osm	2 (1–2)	1–7	0.58 * (0.48–0.67)	0.74	57.5%	81.2%	69	56	13	51	66.1%	1
7-color chart vs. USG	2 (1–2)	1–7	0.68 * (0.60–0.75)	0.83	68.6%	86.2%	70	75	12	32	76.7%	1

8-color Uc chart colors adapted from Armstrong et al. (1994) and 7-color Uc chart was developed by Wardenaar and Bacalzo in 2019 at the Athleat Field Lab. All Spearman correlation coefficients (r) were significant (* = *p* < 0.001) and were based on ranking urine concentration values reported in Table 1 against Uc values presented in Table 2 the 95%CI for *r* is provided in parentheses.

## Data Availability

The underlying research materials related to this paper are available from the corresponding author upon request.
